# Droplet digital PCR quantifies host inflammatory transcripts in feces reliably and reproducibly

**DOI:** 10.1016/j.cellimm.2016.03.007

**Published:** 2016-05

**Authors:** Jennifer Stauber, Nurmohammad Shaikh, M Isabel Ordiz, Phillip I. Tarr, Mark J Manary

**Affiliations:** Department of Pediatrics, Washington University School of Medicine, St. Louis, MO 63110, United States

**Keywords:** Stool, Human mRNA, Quantitative PCR, Stunting, Gut inflammation, Environmental enteric dysfunction

## Abstract

The gut is the most extensive, interactive, and complex interface between the human host and the environment and therefore a critical site of immunological activity. Non-invasive methods to assess the host response in this organ are currently lacking. Feces are the available analyte which have been in proximity to the gut tissue.

We applied a method of concentrating host transcripts from fecal specimens using a existing bead-based affinity separation method for nucleic acids and quantified transcripts using droplet digital PCR (ddPCR) to determine the copy numbers of a variety of key transcripts in the gut immune system. ddPCR compartmentalizes the reaction in a small aqueous droplet suspended in oil, and counts droplets as either fluorescent or non-fluorescent. Glyceraldehyde-3-phosphate dehydrogenase (GAPDH) was used to normalize transcript concentration.

This method was applied to 799 fecal samples from rural Malawian children, and over 20,000 transcript concentrations were quantified. Host mRNA was detected in >99% samples, a threshold for target detection was established at an average expression of 0.02 copies target/GAPDH, above which correlation coefficient between duplicate measurements is >0.95. Quantities of transcript detected using ddPCR were greater than standard qPCR. Fecal sample preservation at the time of collection did not require immediate freezing or the addition of buffers or enzymes. Measurements of transcripts encoding immunoactive proteins correlated with a measure of gut inflammation in the study children, thereby substantiating their relevance. This method allows investigators to interrogate gene expression in the gut.

## Highlights

• Direct measurement of gut epithelial immunology is difficult.
• Fecal host transcript can measured using conservative transcript isolation and droplet digital PCR.
• A new method to non-invasively elucidate gut immunology is described.

## 1 Introduction

Optimal gut health is defined as the ability of the intestines to absorb all necessary dietary nutrients while mounting appropriate inflammatory responses to limit the dissemination of the microbes from the lumen while averting chronic local or systemic inflammation. The many microbes in the gut are thought to modulate gut health. Our awareness of the role of gut microbiota in gut health has increased exponentially in the last 5 years. The most pervasive condition associated with poor gut health worldwide is environmental enteric dysfunction (EED), which is associated with stunting [1], [2], [3]. Stunting affects 25% of the world’s children, whose capacity for physical work, neurocognitive function, linear growth and immunocompetence are compromised [4]. Poor gut health has also been implicated in gastrointestinal tract cancers [5], [6], autoimmune [7], mental health [8], neuro-psychological [9] and cardiovascular [10] disorders.

Unfortunately, direct assessment of gut health is invasive and expensive. The most reliable methods to assess gut health involve direct (endoscopic) visualization of the gut and biopsy, and the dual sugar absorption test [11]. Endoscopy is a resource-intensive procedure that is not well suited to mass screening, or to frequent intra-host assessment. The dual sugar absorption test, also known as the lactulose:mannitol (L:M) test, is administered by orally ingesting a solution of both sugars, and collecting all urine over a timed period of several hours. Lactulose, a disaccharide, is absorbed only through disrupted cell junctions, while mannitol, a monosaccharide, is absorbed across cell membranes and across cell junctions. Once absorbed these sugars are excreted unmetabolized in the urine. Increased L:M is indicative of disrupted architecture of the upper intestinal mucosa and poor gut health [12]. This is a theoretically sound and often used test, but does not provide information on mechanisms underlying increased permeability.

Stool is an easily acquired, but understudied, analyte that contains exfoliated enterocytes, representing gut mucosal tissue. Fecal extractions have been rarely used to analyze expression of individual host transcripts by quantitative PCR (qPCR). qPCR for host fecal transcripts is challenging because human mRNA is estimated to be less than 1% of total fecal RNA, which is predominantly microbial and ribosomal. mRNA in feces is also relatively degraded, and quantification can be further hampered by co-extracted inhibitors.

Here we report an improved methodology to detect fecal host mRNA using droplet digital PCR (ddPCR), as applied to stools from rural Malawian children with varying states of gut health as determined by L:M testing.

## 2 Materials and methods

### 2.1 Fecal samples

Fresh fecal samples were collected from 799 children aged 12–61 months in rural Malawi who participated in one of 3 clinical studies [13], [14], [15]. These children are from families of subsistence farmers, consume water from wells or boreholes and live in unelectrified mud huts. They are at high risk for the enteropathy associated with stunting. All of the subjects completed a carefully conducted L:M test with adequate urine collection and sugar excretion [16].

**Fig. 1:**
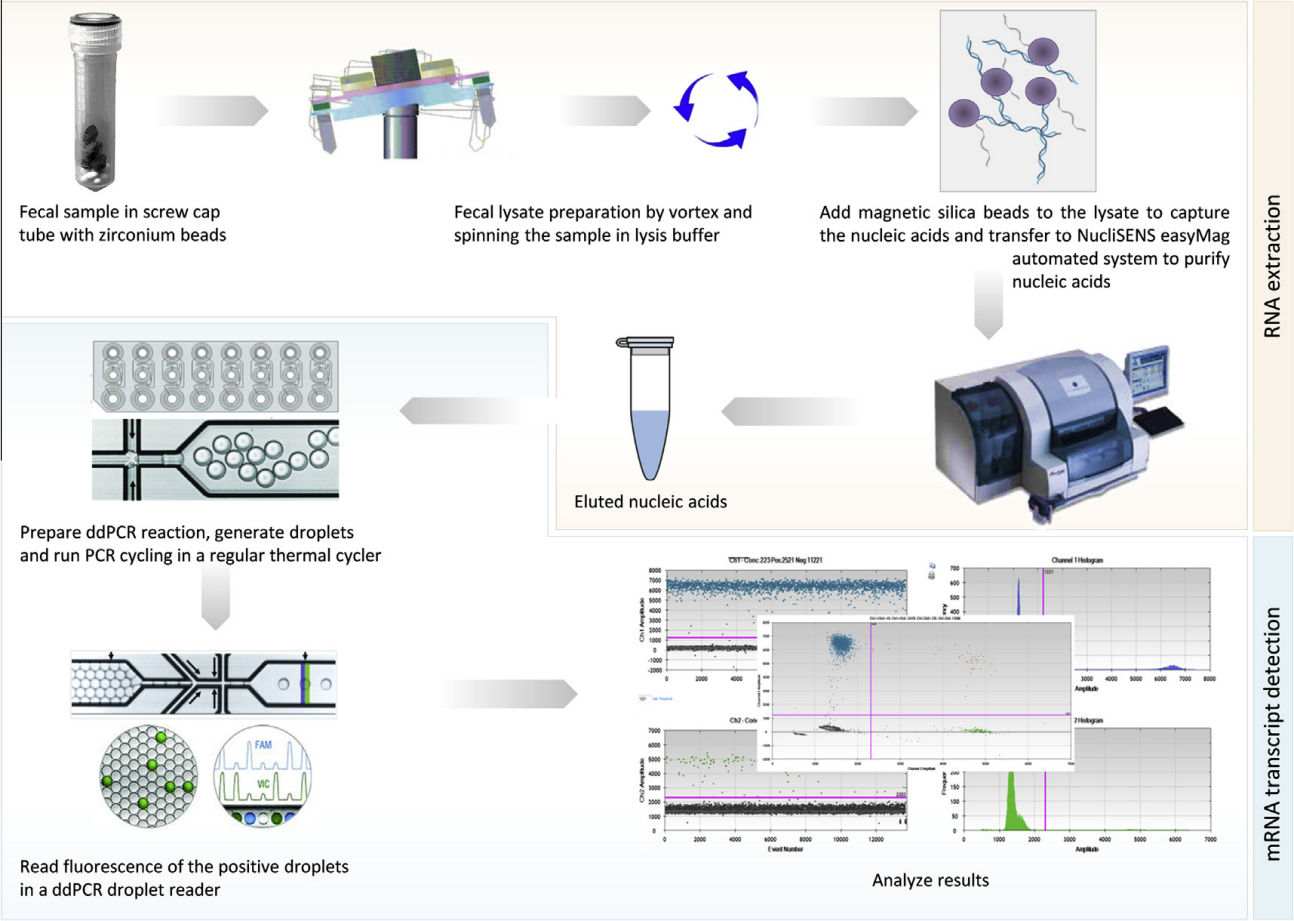
Methodology used to isolate and detect fecal host transcripts.

Fresh stools were collected before completion of the L:M testing using a small, clean non-absorbant, plastic diaper. The stools were immediately transferred to cryovials and flash frozen in liquid nitrogen, without buffers, enzymes or preservative solutions. Samples were transferred to a −80 °C freezer and transported to Washington University (St. Louis, MO), where they were then processed and analyzed for human fecal mRNA as outlined in Fig. 1 and detailed below.

Among the many hundreds of stool samples collected, 16 were divided into 6 aliquots and flash frozen immediately after, and 1, 2, 4, 8 and 24 h after passage. Storage prior to freezing was at ambient (Malawian) temperature, approximately 23–28 °C.

### 2.2 Isolation of fecal RNA

Fecal nucleic acid extractions were prepared using NucliSENS® easyMAG® system (bioMerieux, Durham, NC) and a modified version the protocol of Agapova et al. [17]. Approximately 200–300 mg of frozen stool and 8–10 disruption beads zirconium/silica 23 mm (Research Products International Corp.) was homogenized in 1 ml of easyMAG® lysis buffer using MP FastPrep-24 tissue homogenizer (MP Biomedicals) (60 s, 6.5 m/s,) two times, and then incubated (room temperature, 15 min). Debris was pelleted by centrifugation (10 min, 14,800*g*), and the clarified supernatant was loaded into wells of the easyMAG® cartridges, avoiding visual particulates. EasyMAG® lysis buffer was added to fill the remaining volume to 2 ml, 50 μl NucliSENS® easyMAG® magnetic silica were added. Samples were then extracted following the manufacturer’s instructions for Protocol A using the offboard lysis option.

### 2.3 Droplet digital PCR detection of transcripts

Quantitative polymerase chain reaction assays were performed using duplexed FAM and VIC TaqMan assays in a droplet digital PCR system (QX100; Bio-Rad Laboratories, Inc, Hercules, CA) [18]. Duplicate reactions of 20 μl were prepared using 7.76 μl total nucleic acids, 10 μl ddPCR Supermix for Probes (BioRad), 0.08 μl SuperScript III Reverse Transcriptase (200 U/μl, Invitrogen Corporation, Carlsbad, CA), 0.16 μl RNase OUT (40 U/μl, Invitrogen), and 1 μl of each 20 × TaqMan Gene Expression Assay (Applied Biosystems, Carlsbad, CA). Glyceraldehyde-3-phosphate dehydrogenase (GAPDH) assays were performed for each sample on each plate for normalization of all other targets. PCR reactions were dispersed into droplets using the QX100 droplet generator per the manufacturer’s instruction (BioRad Laboratories) and transferred to a 96-well PCR plate. End point PCR was performed in a C1000 Touch thermal cycler (BioRad) with the following conditions: 50 °C 30′, 95 °C 10′, 40 cycles of 94 °C 30″ followed by 60 °C 1′, 98 °C 10′ and cooling to 4 °C. The fluorescence of each droplet was quantified in the QX100 droplet reader (BioRad Laboratories). DNA extracted from Caco-2 cells was used as a negative control to test the mRNA specificity of each probe analyzed.

### 2.4 Data processing

Absolute quantification was performed in QuantaSoft software (BioRad, Santa Clara, CA). Thresholds were manually set to distinguish between positive and negative droplets. Samples with insufficient negative droplets for a clear baseline were excluded from further analysis. Output from QuantaSoft included concentration and droplet counts for each target in each well. The average concentration for duplicate wells was then calculated and normalized to GAPDH in Access (Microsoft). Final expression results were reported as Target/GAPDH ratios.

### 2.5 Comparison of ddPCR measurements with qPCR

Two fecal nucleic acid extractions were analyzed for interleukin1β (IL1β) and GAPDH by both ddPCR and quantitative real time PCR (qPCR). These 2 samples varied in quantity of human mRNA in the extraction (indicated by GAPDH) and expression of IL1β (indicated by IL1β/GAPDH), the relative quantity of IL1β to GAPDH were chosen to vary by at least an order of magnitude, to represent probes present in both high and low concentrations. Four serial 10-fold dilutions of each sample were analyzed using Taqman assays on both ddPCR (QX100 – BioRad laboratories Inc. Hercules, CA) and qRT-PCR (ABI 7500 Fast Real time PCR system-Life Technologies, Waltham, MA) using equal input quantity of RNA for each dilution. Target copy number calculations were determined for each dilutions and comparisons were made between the concentration of each target detected (copies/μl) as well as the expression of IL1β (IL1/GAPDH).

### 2.6 Correlation of ddPCR measurements with L:M

Normalized expression values for mRNAs were analyzed for Spearman’s correlation to concurrent L:M test results using SPSS (IBM SPSS Statistics for Windows, Version 22.0).

## 3 Results

### 3.1 Human mRNA detection

**Fig. 2:**
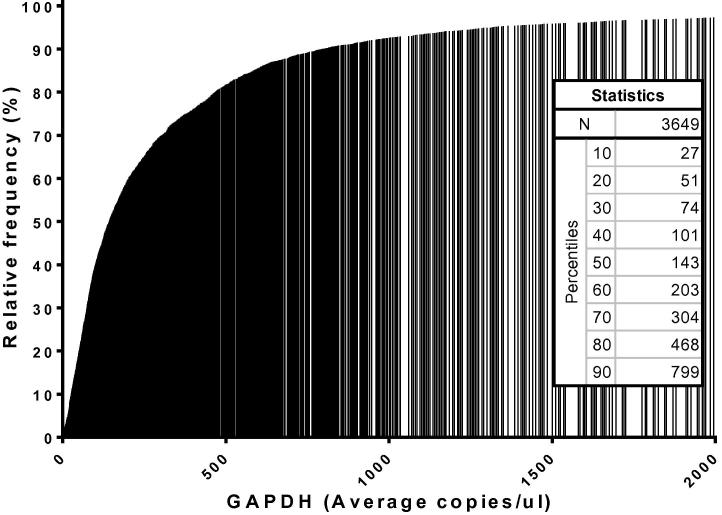
Detectability of human mRNA in fecal samples. GAPDH was assayed by droplet digital PCR in 3649 samples.

This study included analyses of over 20,000 individual host transcript measurements in human feces. Despite the fact that human protein coding targets account for a fraction of a percent of fecal extracts, GAPDH was always detected in our fecal extractions. In 3649 samples assayed for GAPDH in duplicate wells with at least 10,000 droplets/well, the median value was 143 copies/μl GAPDH, with all samples having at least 1 copy/μl. About 80% of the samples assayed had between 25 and 500 copies/μl GAPDH. 292 samples (8%) had GAPDH < 25 copies/μl (Fig. 2). DNA extracted from Caco-2 cells was not detected in any of the samples, indicating that the assay is highly specific for RNA.

### 3.2 mRNA concentration isolated from feces can be estimated reproducibly

**Fig. 3:**
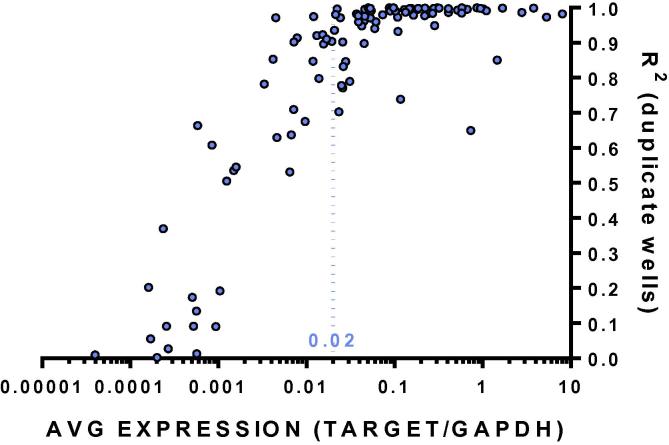
Reproductibility of the measurement of mRNA concentration isolated from feces. The consistency of assay replicates was measured by r^2^ from 107 different mRNA targets assayed in 20 samples/target relative to average expression levels (target/GAPDH).

Data for 107 different mRNA targets assayed in at least 20 samples/target in duplicate wells with a minimum of 10,000 droplets/well show consistency of assay replicates (measured by r^2^) in relation to average expression levels (Target/GAPDH) (Fig. 3).

### 3.3 Comparison of ddPCR and qPCR detection

**Fig. 4:**
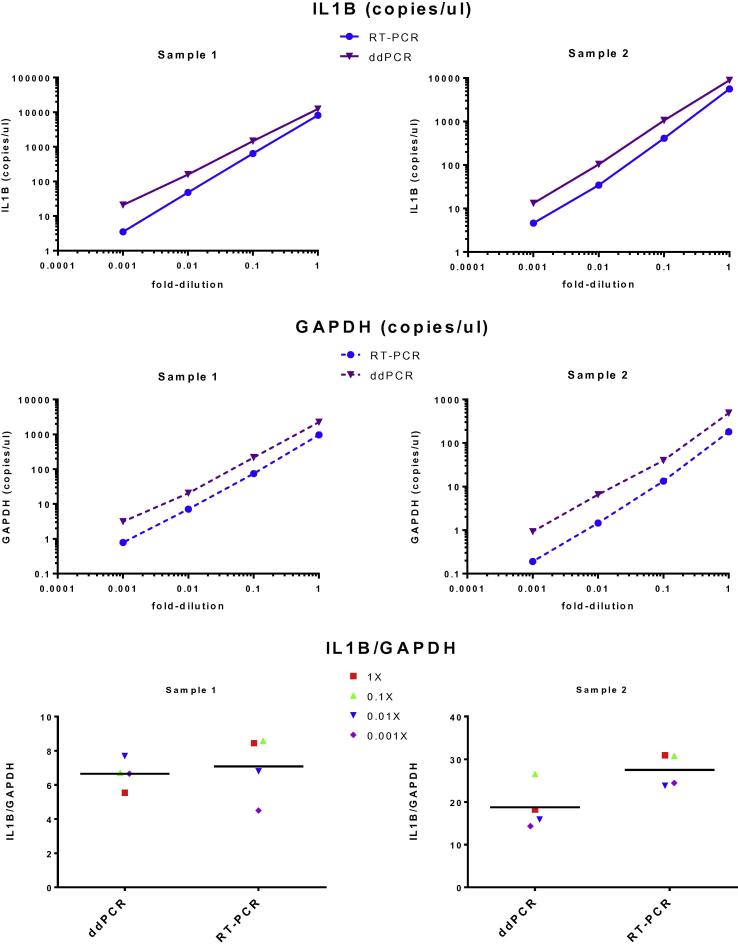
Comparison of ddPCR and qPCR. 2 samples at four different 10-fold dilutions were assayed with (A) IL1B, (B) GAPDH and (C) IL1B/GAPDH ratios were calculated.

Two samples assayed for IL1β and GAPDH in four serial 10-fold dilutions demonstrated a linear relationship over a 1000 fold variation in concentration, and that ddPCR detected more copies/μl of both mRNA targets (Fig. 4). The relative abundance of IL1β to GAPDH in the 2 samples was 6:1 and 19:1 and the ratio of IL-1β/GAPDH were similar on both platforms.

### 3.4 Degradation of mRNA when held at an ambient temperature prior to flash freezing

**Fig. 5:**
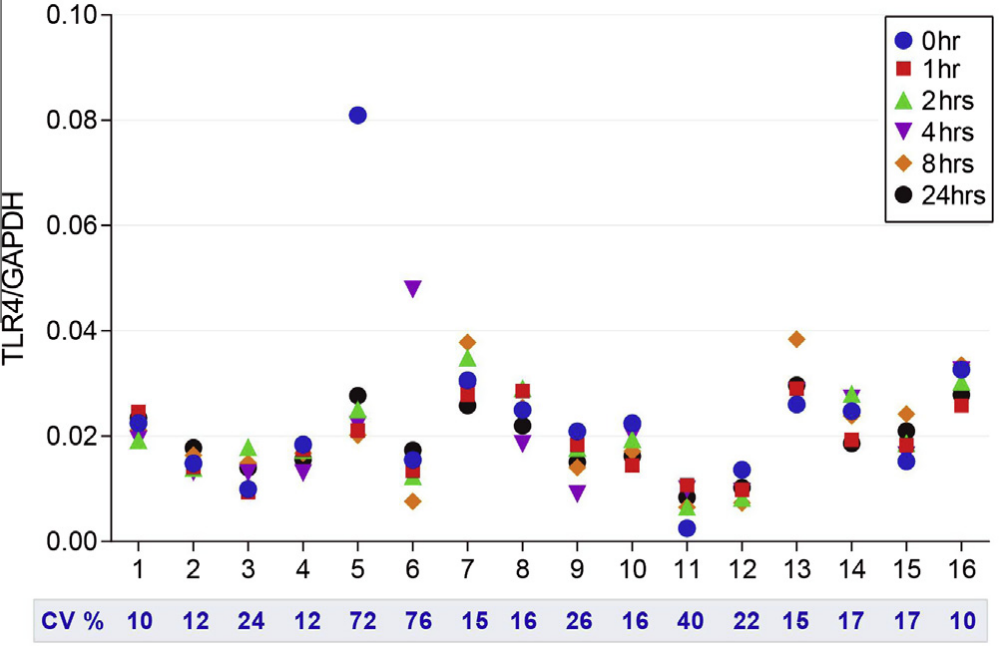
Stability of mRNA when samples are held at room temperature prior to flash freezing. The stability was measured as the TLR-4/GAPDH ratio in 16 samples at 0, 1, 2, 4, 8 and 24 h prior to flash freezing.

GAPDH concentrations were unchanged in identical samples allowed to set at ambient conditions up through 4 h prior to flash freezing, on average they were 336 ± 287 copies/μl, but after 8 h declined to 242 ± 223 copies/μl and to 205 ± 181 copies/μl after 24 h (*P* = 0.04 using a paired *t*-test). Toll-like receptor 4 transcript (TLR4) concentrations for the 16 subjects from whom stool aliquots were frozen after storage at ambient conditions for up to 24 h are portrayed in Fig. 5. The median coefficient of variation in TLR4 measurements was 16%, with 14/16 CV measurements being <26%.

### 3.5 Correlation between ddPCR measures of inflammatory transcripts and L:M measurements

A set of transcripts was chosen for study because of the known association of their cognate proteins with small bowel inflammation, enteric infections or tissue expression in the small bowel, in view of the fact that EED is a small bowel disorder (Supplemental Data 1, Supplemental Data 2). 114/184 (62%) transcripts chosen were detectable in quantities >0.002 copies/copy GAPDH. Among the 53 transcripts tested in at least 70 children, 18 correlated with L:M (Table 1).

## 4 Discussion

We present a robust and reproducible method that uses ddPCR to measure individual host transcripts in feces, in which GAPDH is detected in >99% of thousands of samples tested, and which is more sensitive than qPCR. Moreover, it is not dependent on immediate flash freezing, which is considered the gold standard for stool mRNA preservation [19]. This analytical method successfully identified specific transcripts that are associated with EED, a common cause of gut inflammation in the developing world.

We are able to accurately quantify targets with average expression levels >0.02 target/GAPDH. At this cutoff, 86% of targets have r^2^ values >0.9, as opposed to only 14% of targets with lower expression levels. Therefore, when testing new targets it is advisable first to test a small number of samples and determine average expression before proceeding with large numbers of samples. Extremely low expression targets (<0.001 Target/GAPDH) can still be accurately quantified by concentrating samples or by increasing the number of wells/assay and merging the wells using QuantaSoft software (BioRad, Santa Clara, CA). However these measures may be impractical when routinely assaying large numbers of samples.

We had access to liquid nitrogen for flash freezing fecal samples, but our data show that ambient temperatures for 24 h without preservatives did not permit within sample mRNA degradation. This corroborates the finding of Bennett, et al., in newborn stool (2009). A 34% reduction in GAPDH was seen over the 24 h period, so for samples with intermediate concentrations of GAPDH < 50 copies/μl immediate cryopreservation might be advisable.

The correlations identified between 18 host transcripts and a known gut inflammatory gastrointestinal condition, EED, demonstrate that this method can be used in clinical investigation. A panel of small number of host inflammatory transcripts measured by ddPCR may serve as a surrogate for the L:M test or marker of disease progression. S100A8, which is correlated with L:M, is the host transcript that corresponds to the protein calprotectin; a fecal protein is used to clinically assess bowel inflammation. Calprotectin is one of a very few proteins that can remain intact to allow for reproducible detection as it passes through gastrointestinal tract with its many microbes that secrete proteases. Unlike fecal proteins, about 62% of host transcripts were detected fecal samples. Protein detection in stool has been described as ‘often qualitative and variably sensitive’ [20]. By measuring fecal transcripts, which can be amplified and thus detected when present in smaller quantities, gut health may be more reliably assessed and better understood in the future.

Despite the attributes of this technique, there are some caveats to our findings. This ddPCR method will need to be tested in additional sets of subjects to determine its suitability for detection of fecal host transcripts across other disorders and populations. In samples from healthy adults with good gut health, detection of mRNA might well be more difficult, because of the relative paucity of host transcripts. When measuring transcripts by ddPCR, two probes with different fluorescent labels are placed in the same well. Interactions between probes in the same well may occur and introduce measurement variation. However, we did not observe this among the pairings used. Measurement of host transcripts in feces has previously been reported in a few studies using PCR, but is not in widespread use as analytical method, so this work is an extension of previous investigations [21], [22], [23], [24], [25], [26]. These previous studies were done with a small number of samples, and in many samples no host mRNA was detected. Our method offers improved target detection sensitivity, which we attribute to our isolation method and application of ddPCR. We also recommend setting a minimum threshold of 25 copies/μl of GAPDH in every sample to provide a reliable measurement of targets of interest, most of which are expressed at levels well under that of GAPDH. Assay replicates are highly consistent for mRNA targets with expression levels above 0.02 copies/μl target/GAPDH. We also observed greater numbers of transcript in samples by ddPCR than by qPCR, consistent with previous work [27]. This might be the result of reduced PCR inhibition because of compartmentalization of reactions by the oil medium or by underestimation due to reliance on a standard curve in RT-PCR.

In summary, quantification of host inflammatory transcripts in stool could detect mRNA biomarkers of gut disease. Host transcripts in feces may find application to diagnose conditions where the presence of both certain pathogen and a host inflammatory response is required to produce illness, such as Clostridium difficile infections [26]. Future research is needed to realize this potential.

## Figures and Tables

**Table 1:** Summary of 18 transcripts correlated with L:M.

Gene symbol	Gene function	Transcript Concentration^1^Median (25th, 75th percentiles)	Spearman’s r with L:M	*P* value of r
ACP1	Acts on tyrosine phosphorylated proteins, low-MW aryl phosphates and natural and synthetic acyl phosphates. Isoform 3 does not possess phosphatase activity	0.007 (0.005, 0.011)	−0.104	0.043
AQP9	Forms a channel with a broad specificity. Mediates passage of non-charged solutes including carbamides, polyols, purines, and pyrimidines, whereas amino acids, cyclic sugars, and ions are excluded	0.065 (0.019, 0.150)	0.299	0.01
BIRC3	Regulates caspases and apoptosis, modulates inflammatory signaling and immunity, mitogenic kinase signaling and cell proliferation, as well as cell invasion. Acts as an E3 ubiquitin-protein ligase	0.119 (0.075, 0.192)	−0.125	0.005
CD53	Mediates signal transduction promoting cell development. Complexes with integrins. Mutations in this gene result in immunodeficiency	0.071 (0.031, 0.172)	0.316	0.006
CDX1	Caudal type homeobox 1. Plays a role in the terminal differentiation of the intestine	0.026 (0.016, 0.042)	−0.157	<0.001
DECR1	Auxiliary enzyme of beta-oxidation. It participates in the metabolism of unsaturated fatty enoyl-CoA esters. Catalyzes the NADP-dependent reduction of 2,4-dienoyl-CoA to yield trans-3-enoyl-CoA	0.035 (0.022, 0.047)	0.220	0.043
DEFA6	Has antimicrobial activity against Gram-negative and Gram-positive bacteria. Protects cells against infection with HIV-1	0.038 (0.015, 0.096)	0.138	0.009
HLADRA	Binds peptides derived from antigens that access the endocytic route of antigen presenting cells and presents them on the cell surface for recognition by the CD4 T-cells	0.176 (0.103, 0.279)	−0.136	0.002
IFI30	Lysosomal thiol reductase that reduces protein disulfide bonds. Facilitates the complete unfolding of proteins destined for lysosomal degradation. Plays an important role in antigen processing	0.168 (0.081, 0.288)	0.264	0.024
LYZ	Lysozyme has primarily a bacteriolytic function; those in tissues and body fluids are associated with the monocyte-macrophage system and enhance the activity of immunoagents	0.083 (0.040, 0.137)	0.297	0.011
MUC12	Mucin 12. Codes for key protein in mucous layer. Involved in epithelial cell protection, adhesion modulation, signaling and epithelial cell growth regulation. Stimulated by inflammatory cytokines	0.321 (0.162, 0.541)	−0.271	0.021
PIK3AP1	Signaling adapter that contributes to B-cell development. Links Toll-like receptor signaling to PI3K activation, preventing excessive inflammatory cytokine production. Activates natural killer cells	0.048 (0.022, 0.121)	0.249	0.029
REG1A	Acts as an inhibitor of spontaneous calcium carbonate precipitation. Associated with intestinal, brain and pancreas regeneration	0.040 (0.018, 0.103)	0.188	<0.001
REG3A	Bactericidal C-type lectin which acts exclusively against Gram-positive bacteria and mediates bacterial killing by binding to surface-exposed carbohydrate moieties of peptidoglycan	0.049 (0.019, 0.086)	0.262	0.011
S100A8	Calprotectin, a calcium- and zinc-binding protein which plays a prominent role in the regulation of inflammatory processes and immune response. Induces neutrophil chemotaxis and adhesion	0.386 (0.154, 1.169)	0.096	0.025
SELL	Cell surface adhesion protein. Promotes initial tethering and rolling of leukocytes in endothelia	0.009 (0.003, 0.038)	0.295	0.008
SI	Sucrase isomaltase. A disaccharidase that plays an important role in carbohydrate digestion. Isomaltase activity is specific for α-1,4- and α-1,6-oligosaccharides	0.017 (0.008, 0.036)	−0.100	0.006
TNF	Cytokine that binds to TNFRSF1A/TNFR1. Secreted by macrophages, potent pyrogen, promotes cell death. Induces IL-12 production in dendritic cells	0.004 (0.002, 0.008)	−0.153	<0.001

1 Expressed as copies/copy GAPDH.
