# Supplementary material for: Droplet digital PCR quantifies host inflammatory transcripts in feces reliably and reproducibly
Source: Cell Immunol. 2016 May;303:43–9. doi: 10.1016/j.cellimm.2016.03.007 (PMC4863679; doi:10.1016/j.cellimm.2016.03.007)
Supplement: Supplementary data 1 and 2 [file mmc1.pdf]

Supplementary Data 1. List of 114 transcripts tested and detected by droplet digital PCR.

Gene symbol	Description	Mean ± SD (n)
**ACP1**	Acid Phosphatase 1, Soluble	0.009 ± 0.009 (378)
**ACSL1**	Acyl-CoA Synthetase Long-Chain Family Member 1	0.857 ± 2.160 (39)
**ADI1**	Acireductone Dioxygenase 1	0.090 ± 0.038 (5)
**ALDH18A1**	Aldehyde Dehydrogenase 18 Family, Member A1	0.016 ± 0.012 (40)
**AQP9**	Aquaporin 9	0.145 ± 0.318 (73)
**ARG2**	Arginase 2	0.007 ± 0.005 (63)
**B2M**	Beta-2-Microglobulin	7.902 ± 3.232 (29)
**BCL2A1**	BCL2-Related Protein A1	0.660 ± 0.800 (39)
**BIRC3**	Baculoviral IAP Repeat Containing 3	0.166 ± 0.179 (512)
**CA13**	Carbonic Anhydrase XIII	0.034 ± 0.040 (14)
**CCL20**	Chemokine (C-C Motif) Ligand 20	0.305 ± 0.243 (211)
**CD53**	CD53 Molecule	0.144 ± 0.214 (73)
**CD69**	CD69 Molecule	0.053 ± 0.085 (157)
**CDH1**	Cadherin 1, Type 1	0.300 ± 0.177 (66)
**CDX1**	Caudal Type Homeobox 1	0.048 ± 0.355 (534)
**CISD2**	CDGSH Iron Sulfur Domain 2	0.028 ± 0.025 (132)
**CLCA4**	Chloride Channel Accessory 4	2.825 ± 2.338 (52)
**CLDN15**	Claudin 15	0.015 ± 0.019 (361)
**CMTM6**	CKLF-Like MARVEL Transmembrane Domain Containing 6	0.045 ± 0.055 (116)
**CPEB4**	Cytoplasmic Polyadenylation Element Binding Protein 4	0.138 ± 0.244 (54)
**CPNE8**	Copine VIII	0.002 ± 0.026 (90)
**CR1**	Complement Component (3b/4b) Receptor 1 (Knops Blood Group)	0.018 ± 0.063 (38)
**CSF3R**	Colony Stimulating Factor 3 Receptor (Granulocyte)	0.087 ± 0.246 (25)
**DAT**	Solute Carrier Family 6 (Neurotransmitter Transporter), Member 3	0.026 ± 0.007 (5)
**DECR1**	2,4-Dienoyl CoA Reductase 1, Mitochondrial	0.041 ± 0.027 (85)
**DEFA5**	Defensin, Alpha 5, Paneth Cell-Specific	0.164 ± 0.212 (153)
**DEFA6**	Defensin, Alpha 6, Paneth Cell-Specific	0.083 ± 0.135 (358)
**DEFB1**	Defensin, Beta 1	0.013 ± 0.017 (554)
**DNAJC10**	DnaJ (Hsp40) Homolog, Subfamily C, Member 10	0.007 ± 0.006 (51)
**DST**	Dystonin	0.156 ± 0.197 (48)
**E2F4**	E2F Transcription Factor 4, P107/P130-Binding	0.043 ± 0.028 (26)
**EMR2**	Adhesion G Protein-Coupled Receptor E2	0.006 ± 0.010 (65)
**EPCAM**	Epithelial Cell Adhesion Molecule	0.149 ± 0.176 (25)
**ETV3**	Ets Variant 3	0.048 ± 0.031 (25)
**EZH1**	Enhancer Of Zeste 1 Polycomb Repressive Complex 2 Subunit	0.020 ± 0.027 (33)
**EZH2**	Enhancer Of Zeste 2 Polycomb Repressive Complex 2 Subunit	0.008 ± 0.011(21)
**FAM65B**	Family With Sequence Similarity 65, Member B	0.056 ± 0.195 (544)
**FCAR**	Fc Fragment Of IgA Receptor	0.048 ± 0.032 (14)
**GAA**	Glucosidase, Alpha; Acid	0.011 ± 0.007 (6)
**GCH1**	GTP Cyclohydrolase 1	0.026 ± 0.020 (298)
**GCHFR**	GTP Cyclohydrolase I Feedback Regulator	0.006 ± 0.001 (6)
**GJB2**	Gap Junction Protein, Beta 2, 26kDa	0.036 ± 0.022 (19)
**GPR108**	G Protein-Coupled Receptor 108	0.027 ± 0.014 (33)
**HK2**	Hexokinase 2	0.158 ± 0.126 (44)
**HLA-C**	Major Histocompatibility Complex, Class I, C	1.380 ± 1.091 (18)
**HLA-DRA**	Major Histocompatibility Complex, Class II, DR Alpha	0.221 ± 0.169 (545)
**ICAM1**	Intercellular Adhesion Molecule 1	0.017 ± 0.035 (53)
**IFI30**	Interferon, Gamma-Inducible Protein 30	0.309 ± 0.611 (73)
**IFI6**	Interferon, Alpha-Inducible Protein 6	0.078 ± 0.144 (85)
**IL1B**	Interleukin 1, Beta	1.398 ± 2.069 (165)
**IL1RN**	Interleukin 1 Receptor Antagonist	0.906 ± 1.499 (43)
**IL4R**	Interleukin 4 Receptor	0.134 ± 0.141 (79)
**IL8**	Chemokine (C-X-C Motif) Ligand 8	1.519 ± 2.627 (206)
**KRT20**	Keratin 20, Type I	2.924 ± 2.136 (154)
**LAP3**	Leucine Aminopeptidase 3	0.118 ± 0.113 (724)
**LAPTM5**	Lysosomal Protein Transmembrane 5	0.507 ± 1.098 (39)

Gene symbol	Description	Mean ± SD (n)
**LCN2**	Lipocalin 2	0.578 ± 0.472 (507)
**LCP1**	Lymphocyte Cytosolic Protein 1 (L-Plastin)	0.221 ± 0.490 (33)
**LCT**	Lactase	0.106 ± 1.877 (651)
**LMO7**	LIM Domain 7	0.590 ± 0.495 (33)
**LPP**	LIM Domain Containing Preferred Translocation Partner In Lipoma	0.039 ± 0.023 (6)
**LUC7L3**	LUC7-Like 3 Pre-MRNA Splicing Factor	0.252 ± 0.290 (44)
**LYZ**	Lysozyme	0.131 ± 0.181 (73)
**MAP4K4**	Mitogen-Activated Protein Kinase Kinase Kinase Kinase 4	0.014 ± 0.024 (152)
**MARK2**	MAP/Microtubule Affinity-Regulating Kinase 2	0.058 ± 0.037 (128)
**MGAM**	Maltase-Glucoamylase	0.035 ± 0.082 (62)
**MNDA**	Myeloid Cell Nuclear Differentiation Antigen	0.393 ± 0.627 (45)
**MUC12**	Mucin 12, Cell Surface Associated	0.376 ± 0.263 (72)
**MUC2**	Mucin 2, Oligomeric Mucus/Gel-Forming	2.576 ± 2.978 (23)
**MUC20**	Mucin 20, Cell Surface Associated	0.180 ± 0.142 (24)
**MUC21**	Mucin 21, Cell Surface Associated	0.002 ± 0.004 (24)
**MUC4**	Mucin 4, Cell Surface Associated	0.195 ± 0.238 (28)
**NFYC**	Nuclear Transcription Factor Y, Gamma	0.010 ± 0.007 (18)
**NQO2**	NAD(P)H Dehydrogenase, Quinone 2	0.022 ± 0.015 (33)
**OAT**	Ornithine Aminotransferase	0.017 ± 0.018 (370)
**OCLN**	Occludin	0.245 ± 0.166 (35)
**PIGA**	Phosphatidylinositol Glycan Anchor Biosynthesis, Class A	0.010 ± 0.007 (16)
**PIGR**	Polymeric Immunoglobulin Receptor	5.210 ± 2.642 (56)
**PIK3AP1**	Phosphoinositide-3-Kinase Adaptor Protein 1	0.199 ± 0.420 (77)
**PLEK**	Pleckstrin	3.762 ± 7.734 (39)
**PPCDC**	Phosphopantothenoylcysteine Decarboxylase	0.007 ± 0.005 (20)
**PPP4R1**	Protein Phosphatase 4, Regulatory Subunit 1	0.038 ± 0.041(103)
**RBM25**	RNA Binding Motif Protein 25	0.287 ± 1.262 (77)
**REG1A**	Regenerating Islet-Derived 1 Alpha	0.113 ± 0.272 (598)
**REG1B**	Regenerating Islet-Derived 1 Beta	0.088 ± 0.247 (615)
**REG3A**	Regenerating Islet-Derived 3 Alpha	0.094 ± 0.143 (94)
**RIC8A**	RIC8 Guanine Nucleotide Exchange Factor A	0.014 ± 0.012 (22)
**RORC**	RAR-Related Orphan Receptor C	0.007 ± 0.009 (54)
**S100A12**	S100 Calcium Binding Protein A12	0.024 ± 0.039 (68)
**S100A8**	S100 Calcium Binding Protein A8	0.979 ± 1.738 (550)
**SDHA**	Succinate Dehydrogenase Complex, Subunit A, Flavoprotein (Fp)	0.028 ± 0.014 (32)
**SELL**	Selectin L	0.052 ± 0.134 (80)
**SERPINA1**	Serpin Peptidase Inhibitor, Clade A (Alpha-1 Antiproteinase, Antitrypsin), Member 1	0.501 ± 0.481 (90)
**SI**	Sucrase-Isomaltase (Alpha-Glucosidase)	0.115 ± 0.899 (747)
**SLC25A40**	Solute Carrier Family 25, Member 40	0.006 ± 0.006 (99)
**SLC26A3**	Solute Carrier Family 26 (Anion Exchanger), Member 3	1.446 ± 1.394 (23)
**SLC2A3**	Solute Carrier Family 2 (Facilitated Glucose Transporter), Member 3	0.279 ± 0.725 (23)
**SNAI2**	Snail Family Zinc Finger 2	0.008 ± 0.016 (5)
**SOD2**	Superoxide Dismutase 2, Mitochondrial	0.986 ± 1.241 (197)
**SORL1**	Sortilin-Related Receptor, L(DLR Class) A Repeats Containing	0.299 ± 1.112 (33)
**SRGN**	Serglycin	0.942 ± 1.498 (74)
**STXBP5**	Syntaxin Binding Protein 5 (Tomosyn)	0.015 ± 0.008 (44)
**STYK1**	Serine/Threonine/Tyrosine Kinase 1	0.020 ± 0.017 (122)
**TAGAP**	T-Cell Activation RhoGTPase Activating Protein	0.573 ± 1.857 (73)
**TFRC**	Transferrin Receptor	0.005 ± 0.003 (16)
**TGFB1**	Transforming Growth Factor, Beta 1	0.037 ± 0.030 (21)
**TIMP1**	TIMP Metallopeptidase Inhibitor 1	0.038 ± 0.050 (131)
**TJP1**	Tight Junction Protein 1	0.049 ± 0.054 (85)
**TLR4**	Toll-Like Receptor 4	0.033 ± 0.044 (746)
**TNF**	Tumor Necrosis Factor	0.008 ± 0.015 (747)
**TOM1**	Target Of Myb1 Membrane Trafficking Protein	0.058 ± 0.055 (97)
**TRIM8**	Tripartite Motif Containing 8	0.027 ± 0.017 (56)
**XIAP**	X-Linked Inhibitor Of Apoptosis, E3 Ubiquitin Protein Ligase	0.023 ± 0.032 (90)

Supplemental Data 2. Listing of 70 transcripts evaluated by droplet digital PCR and found to have a majority of the measurements below the threshold of reliable detection, < 0.002 copies/copy GAPDH.

Gene Symbol	Average EXPRESSION	StDev EXPRESSION	Min EXPRESSION	Max EXPRESSION	Number Samples Analyzed
**ALDH3B1**	0.00281	0.00227	0.00091	0.00784	14
**ARHGAP31**	0.00151	0.00063	0.00055	0.00209	5
**ATP1A2**	0.00006	0.00009	0.00000	0.00018	5
**CCDC110**	0.00060	0.00109	0.00000	0.00254	5
**CCL1**	0.00021	0.00038	0.00000	0.00097	6
**CCR9**	0.00010	0.00016	0.00000	0.00058	34
**CD163**	0.00037	0.00087	0.00000	0.00429	38
**CD3G**	0.00062	0.00097	0.00000	0.00409	50
**CD4**	0.00091	0.00084	0.00000	0.00286	15
**CHST1**	0.00011	0.00024	0.00000	0.00053	5
**CLDN2**	0.00002	0.00015	0.00000	0.00085	34
**CP**	0.00012	0.00017	0.00000	0.00037	5
**CTNNA3**	0.00000	0.00000	0.00000	0.00000	6
**DCDC2**	0.00000	0.00000	0.00000	0.00000	5
**ESYT1**	0.00269	0.00317	0.00000	0.01287	18
**ETV2**	0.00007	0.00012	0.00000	0.00031	6
**FAT2**	0.00059	0.00083	0.00000	0.00178	6
**FGF8**	0.00012	0.00014	0.00000	0.00043	15
**FOXP3**	0.00127	0.00261	0.00000	0.00930	16
**HOXB8**	0.00074	0.00229	0.00000	0.01184	27
**IFNg**	0.00317	0.00633	0.00000	0.04254	88
**IGJ**	0.00052	0.00069	0.00000	0.00274	57
**IL10**	0.00054	0.00072	0.00000	0.00302	54
**IL12A**	0.00073	0.00108	0.00000	0.00477	55
**IL13**	0.00070	0.00257	0.00000	0.01782	50
**IL17A**	0.00045	0.00128	0.00000	0.01080	89
**IL17B**	0.00008	0.00018	0.00000	0.00044	6
**IL17F**	0.00102	0.00544	0.00000	0.05096	92
**IL17RB**	0.00107	0.00104	0.00000	0.00264	6
**IL17RE**	0.00008	0.00019	0.00000	0.00046	6
**IL17REL**	0.00102	0.00063	0.00043	0.00192	6
**IL22**	0.00132	0.00227	0.00000	0.01817	152
**IL22RA2**	0.00052	0.00083	0.00000	0.00312	21
**IL23A**	0.00171	0.00255	0.00000	0.02359	428
**IL25**	0.00009	0.00020	0.00000	0.00073	22
**IL4**	0.00029	0.00096	0.00000	0.00644	76
**IL6**	0.00240	0.00361	0.00000	0.01541	19
**IL9**	0.00115	0.00506	0.00000	0.03660	114
**IQCB1**	0.00109	0.00121	0.00000	0.00248	6
**JAKMP3**	0.00000	0.00000	0.00000	0.00000	5
**KCNH1**	0.00004	0.00010	0.00000	0.00022	5
**KIFAP3**	0.00145	0.00079	0.00031	0.00237	5
**MADCAM1**	0.00073	0.00159	0.00000	0.00659	42

Assay	Average EXPRESSION	StDev EXPRESSION	Min EXPRESSION	Max EXPRESSION	Number Samples Analyzed
**MAP3K1**	0.00376	0.00263	0.00103	0.00750	6
**MDH1B**	0.00013	0.00019	0.00000	0.00046	6
**MID2**	0.00070	0.00055	0.00020	0.00155	6
**MPO**	0.00169	0.00381	0.00000	0.03205	163
**MT4**	0.00005	0.00009	0.00000	0.00027	30
**MYLK**	0.00258	0.00163	0.00000	0.00674	39
**NOD1**	0.00011	0.00028	0.00000	0.00069	6
**NOD2**	0.00219	0.00300	0.00000	0.00801	6
**NR1H3**	0.00328	0.00259	0.00051	0.01240	27
**OTC**	0.00078	0.00092	0.00000	0.00373	40
**PRODH**	0.00016	0.00044	0.00000	0.00332	107
**PSEN2**	0.00432	0.00392	0.00035	0.01694	20
**RAD51**	0.00070	0.00102	0.00000	0.00247	5
**RASL10A**	0.00050	0.00029	0.00014	0.00087	5
**RBFOX1**	0.00008	0.00014	0.00000	0.00032	5
**REG3G**	0.00013	0.00069	0.00000	0.00406	34
**REG4**	0.00443	0.00401	0.00000	0.02833	204
**SIGLEC8**	0.00050	0.00120	0.00000	0.00526	30
**SLC6A3**	0.00000	0.00000	0.00000	0.00000	5
**SOGA1**	0.00094	0.00052	0.00036	0.00168	6
**SRD5A2**	0.00000	0.00000	0.00000	0.00000	5
**SYCP2**	0.00000	0.00001	0.00000	0.00002	14
**TBC1D8B**	0.00237	0.00163	0.00046	0.00678	20
**TBX21**	0.00310	0.01422	0.00000	0.10616	60
**TREH**	0.00144	0.00203	0.00000	0.01343	51
**USP6**	0.00012	0.00019	0.00000	0.00046	5
**ZMAT3**	0.00275	0.00223	0.00030	0.00749	13
